# A new approach to overcoming resistance to immunotherapy: nanotechnology

**DOI:** 10.3389/fonc.2023.1210245

**Published:** 2023-08-10

**Authors:** Jiangbo Shao, Ying Jin, Chunxiang Jin

**Affiliations:** ^1^ Department of Ultrasound, China–Japan Union Hospital of Jilin University, Changchun, Jilin, China; ^2^ Department of Breast Surgery, The First Hospital of Jilin University, Changchun, Jilin, China

**Keywords:** cancer, microenvironment, immunotherapy, drug resistance, nanotechnology

## Abstract

Immunotherapy for immune response has ushered in a new era of cancer treatment. In recent years, new immunotherapeutic agents have been introduced into clinical trials and even approved for marketing. However, the widespread use of immunotherapeutic agents faces an unavoidable challenge: immunotherapy does not work at all for some patients, or has good efficacy in the initial phase, but immunotherapy resistance develops within a short period of time, and immunotherapy can also cause serious adverse effects such as autoimmune inflammation and non-specific inflammation. How to enable patients to overcome drug resistance, reduce the toxic side effects of drugs, enhance patient compliance and improve patient survival has become a problem that clinicians have to face. The advent of nanotechnology provides an encouraging platform for immunotherapy. It can not only improve the bioavailability and stability of drugs and reduce toxic side effects, but also reduce resistance to immunotherapy. Here, we discuss these research advances and discuss potential challenges and future directions.

## Introduction

In 2013, tumor immunotherapy was named the most important scientific breakthrough of the year by the journal Science for its outstanding efficacy and innovation (1). Immunotherapy has now become an important treatment for many malignant tumors, ushering in a new era of tumor treatment and bringing light to the fight against tumors. However, as the use of immunotherapy has become more popular, the problem of drug resistance to immunotherapy has gradually emerged. Due to its special mechanism of action, the mechanism of resistance to immunotherapy is different from that of resistance to conventional chemotherapy (2–6), so how to overcome resistance to immunotherapy is currently an urgent challenge to be solved.

Recent advances in nanomedicine have led to a plethora of new nanomedicines targeting immunotherapeutic targets (7–11). Furthermore, as research has progressed, it has been found that nanomedicines not only have the ability to target drug delivery, but also have the ability to remodel the immune microenvironment, thereby enhancing the efficacy of anti-tumor immunotherapy (12). Therefore, we believe that nanomedicines provide a relevant reference for basic research and the development of improved clinical treatment regimens, and are expected to be an important weapon in overcoming drug resistance in immunotherapy. In this article, we review the mechanisms of immunotherapy resistance and summarize the recent research advances in nanomedicine with respect to the mechanisms of resistance, with the aim of providing clues for overcoming immunotherapy resistance.

## Mechanisms of immune resistance

Tumors are inherently complex and heterogeneous, and resistance involves not only the tumor but also complex metabolic, inflammatory and neovascular mechanisms, many of which are still unknown. At present, the mechanisms of resistance to immunotherapy can be divided into intrinsic and extrinsic mechanisms. Intrinsic mechanisms of tumor immune resistance include alterations in anti-tumor immune response pathways and alterations in tumor cell signaling leading to a suppressive immunosuppressive microenvironment. External factors mainly include those associated with the local tumor microenvironment. The tumor immune microenvironment is a complex network of interactions involving tumor and various non-tumor cells, including fibroblasts, macrophages, B/T lymphocytes and antigen presenting cells (APCs), which in turn affect the whole body.

### Aspects of tumor cell

#### APCs

The immune response pathway involves the processing of tumor-associated antigenic peptides by antigen-presenting cells (APCs), presentation to CD8^+^ T cells, stimulation of T cell proliferation and activation, and activation of T cells to kill tumor cells in the TME (13). Any alteration in the antitumor immune response pathway has the potential to lead to resistance to immunotherapy.

High tumor mutational burden (TMB) (non-synonymous mutations), microsatellite instability (MSI) and defective mismatch repair (dMMR) are intrinsic tumor features associated with anti-tumor immune responses and responses to immune checkpoint inhibitors (ICIs) (14, 15). These responses are closely associated with increased generation of tumor-associated antigens (TAA) and tumor-specific antigens (TSA), with neoantigens conferring greater immunogenicity and increased T-cell infiltration, as has been demonstrated in a variety of tumors (16–19). However, tumor cells tend to suppress T-cell activation by reducing or losing antigen expression (20) and regulate autoantigenicity by endocytosis of antigens or antigen shedding to mediate immune escape. In addition, the host can selectively eliminate TSA-expressing cells and, to some extent, promote the production of tumor antigen-losing variants (21). Tumor cells, like viruses, can also undergo ‘antigenic drift’, resulting in epitope mutations that alter tumor antigenicity and thus evade T cell-mediated attack (22). Endoplasmic reticulum (ER) stress and autophagy determine the immunogenicity of tumor cell death (23, 24). The high presence of LC3b puncta in the cytoplasm of tumor cells represents an active autophagic mechanism within tumor cells and is associated with the infiltration of tumor-infiltrating lymphocytes (TILs) and a favorable clinical outcome, whereas tumor cells do not respond to ER stress or autophagy induction may lead to resistance to ICIS (25, 26).

#### Tumor cell-associated pathways

Deletion of the phosphatase and tensin homologue (PTEN) gene on chromosome 10 has been shown to be directly involved in the regulation of antitumor immunity. First, there is a significant correlation between loss of PTEN and reduced T-cell infiltration, ultimately leading to resistance to PD-1 monoclonal antibodies (27–29). In addition, dysfunctional PTEN also promotes the aggregation of suppressor immune cells, such as myeloid-derived suppressor cells (MDSCs) and regulatory T cells (Tregs) (30–33). More importantly, deletion of PTEN expression has been shown to down-regulate autophagy (34, 35), which can effectively support tumor progression.

In addition, alterations associated with the IFN-γ pathway can also affect immune resistance. In patients receiving immunotherapy, tumor cells can downregulate or alter the IFN-γ pathway, such as loss-of-function alleles in the gene encoding JAK1/2 and alterations in STAT1, to evade the effects of IFN-γ and thus develop resistance (36, 37). Zaretsky et al (38) showed that melanoma patients resistant to PD-1 treatment acquired loss-of-function mutations in JAK1/2. Although tumor cells were still recognized by T cells, their JAK1/2 mutation rendered them insensitive to the anti-proliferative effects of IFN-γ and they lacked IFN-γ-induced PD-L1 and MHC class I surface expression. Similarly, analysis of tumors from ipilimumab-refractory patients showed that mutations in the IFN-γ pathway genes IFNGR1/2, JAK1/2 and IRF1 inhibit the response of tumor cells to IFN-γ signaling (39). This facilitates tumor escape from T-cell immunity, thereby conferring resistance to anti-CTLA4 therapy.

### Microenvironment

#### Immune checkpoint

Checkpoint blockade with specific mAbs helps to inhibit pathways that maintain the duration and strength of the immune system. Inhibition of these checkpoint molecules works by reinvigorating the adaptive immune system and selectively eliminating primary and metastatic tumors. Currently, cytotoxic T lymphocyte-associated antigen-4(CTLA4), programmed death receptor-1(PD-1) and its ligand(PD-L1) are the main targets of immunotherapy and are the most widely used in clinical practice (40). In one study, RNA sequencing analysis of patients with NSCLC resistant to PD-L1 inhibitors demonstrated the presence of PD-L1 variant fragments in patients and confirmed their inhibitory effect on T-cell activity (37). TIM-3 (T cell immunoglobulin domain and mucin domain-3) is a negatively regulated immune checkpoint expressed on the surface of T cells, Treg cells and other innate immune cells and is capable of causing T cell depletion in cancer and chronic viral infections. It has been shown that T cells co-expressing TIM-3/PD-1 are more likely to fail, and that patients who fail to respond to anti-PD-1 therapy are often highly correlated with TIM-3 expression, while patients who are resistant to PD-1 therapy are also due to selective overexpression of TIM-3, resulting in tumor immune escape (41). Drugs and drug combinations targeting TIM-3 are therefore being developed to overcome immunotherapy resistance.

#### TAMs

Tumor-associated macrophages (TAMs) have also been implicated in patient response to immunotherapy (42, 43).TAMs are classified into M1 and M2 macrophages based on their different activation pathways and expression of surface molecules: M1 type macrophages promote anti-tumor immunity and M2 type macrophages promote tumor progression by supporting angiogenesis, tumor cell metastasis and suppression of effector CD8^+^ T cells and NK cells due to reduced efficiency of antigen presentation (44–46). Recruitment of TAMs to tumor sites is mediated by tumor-derived proteins (e.g. CSF-1, VEGF and chemokines). The recruitment of TAMs to tumor sites is mediated by tumor-derived effector proteins such as CSF-1, VEGF and chemokines (47). tumors (48–51). A higher density of TAMs is associated with a poor clinical prognosis in cancer patients (52, 53). Fritz et al (54) found that depletion of TAMs can reduce tumor growth in a mouse model of lung adenocarcinoma through downregulation of M2 macrophages. It has been suggested that inactivation of CCL2 and/or CCR2 signaling is responsible for this phenomenon. Similar findings have been reported in other cancer types (e.g. T-cell lymphoma (55), colorectal cancer (56), lung cancer and breast cancer (56–58), where suppression or elimination of these macrophages in the TME may improve patient prognosis.

#### MDSCs

MDSCs contain a panel of neutrophils and monocytes with potent immunosuppressive properties that mediate immune responses induced by T cells, B cells and NK cells (59). Higher levels of neutrophils within the tumor are negatively associated with clinical outcome in cancer patients (60). Indeed, using multidimensional imaging, Si et al (61) provided direct evidence that MDSCs inhibit the expression of Teff-secreted granzyme B and Ki67 (markers of Teff cytotoxicity and proliferation, respectively). The presence of MDSCs in TME is strongly correlated with the efficacy of immunotherapy, as blockade of these cells leads to improved preclinical (62) and clinical outcomes (63). Due to the important role of MDSCs in promoting angiogenesis, tumor invasion and metastasis, these cells have become therapeutic targets for cancer (59, 64–68).

#### CAFs

Cancer-associated fibroblasts (CAFs) are diverse stromal cell populations with multiple functions, including stromal deposition and remodeling, crosstalk with infiltrating leukocytes, and interaction with cancer cells (69). The presence and role of activated CAFs in the microenvironment has been associated with poor prognosis in several cancers (70). CAFs suppress the immune system by promoting physical and chemical barriers. CAFs induce a TH2 phenotype in neighboring cells, and cytokines produced by TH2 cells induce myeloid differentiation into MDSCs (71). CAFs also induce monocytes to differentiate into M2-type TAMs (72, 73), produce fibronectin and secrete TGF-β and IDO (74), further indicating the expansion of Treg cells. In addition, CAFs inhibit the activity of CD8^+^ T cells by expressing PD-L1 (75, 76).

#### NK cells

NK cells can facilitate the recruitment of DC to solid tumors by releasing CC-chemokine ligand 5 (CCL5), XC-chemokine ligand 1 (XCL1) and XCL2. In addition, NK cells enter lymph nodes and influence the T cell response; and through the regulation of antigen-presenting cells, thereby regulating T cells. Activated NK cells can kill immature DCs while retaining mature DCs, thus ensuring successful T cell priming. Thus, the manipulation of NK cells in cancer aims to initiate a multilayered immune response, ultimately leading to protective and long-lasting immunity against the tumor (77).

In recent years, NK cell-associated immunotherapy has emerged as an alternative to ICB-based or vaccine-based immunotherapy (78–81). However, its therapeutic effects are largely limited by the downregulation of recognition ligands, and its immune effects can be further blocked by the secretion of tumor microenvironment such as transforming growth factor-β (TGF-β) (82–84). Researchers have demonstrated the benefits of combining immunotherapy with chemotherapy in cancer treatment (85–87). Therefore, there is great interest in developing combined strategies to enhance NK cell immunity.

#### Immune-related cytokines

Interferon gamma is a cytokine produced and secreted by effector T cells (TEFFs) and APCs. It acts through the JAK-STAT pathway (36) and has a dual role in anti-tumor immunity. Interferon γ can induce the production of the chemokines CXCL9 and CXCL10 and promote the recruitment of CXCR3^+^ lymphocytes and other immune cells around tumor cells, thereby exerting an anti-tumor immune effect (88); it can also exert direct anti-tumor cell proliferation and pro-apoptotic effects by binding to cell surface receptors and triggering a series of events that inhibit tumor cell growth and promote tumor cell death (89); In addition, IFN-γ can play a role in immune escape by increasing the expression of PD-L1 on the surface of tumor cells (89), and therefore altered IFN-γ secretion is thought to be closely associated with immune drug resistance.

#### Tregs

Tregs are an important subset of T cells that help prevent excessive immune responses and autoimmunity, and can infiltrate human tumors and promote tumor growth (90). These FoxP3-expressing cells inhibit the Teff response either directly through physical contact or indirectly by suppressing the secretion of inhibitory cytokines including IL-10, IL-35 and TGF-β (91–94). After anti-CTLA-4 mAb treatment, the ratio of Teffs to Tregs was positively correlated with treatment response depending on the presence of macrophages expressing the Fcγ receptor (95), and the use of anti-CTLA4 antibodies with an enhanced Fcγ R binding profile is recommended to achieve robust anti-tumour responses and improved survival (96). In a clinical trial using ipilimumab to treat patients with advanced melanoma, increased TIL was found to be associated with better outcomes (97). A clinical follow-up study showed that although anti-CTLA-4 immunotherapy promoted intra-tumoral Teff infiltration, it did not lead to FoxP3 T-cell depletion in human cancers (98). These seminal studies on the balance between Teffs and Tregs suggest that increased numbers of tumour-infiltrating Teffs, rather than depletion of Tregs, can be used to predict sensitivity to anti-CTLA-4 immunotherapy. If the ratio of these two T cell subpopulations responding to immunotherapy in TME is unfavorable to Teffs, resistance may emerge throughout the course of treatment.

## Treatment

### Tumor cell aspects

#### Targeting APCs

**Figure 1:**
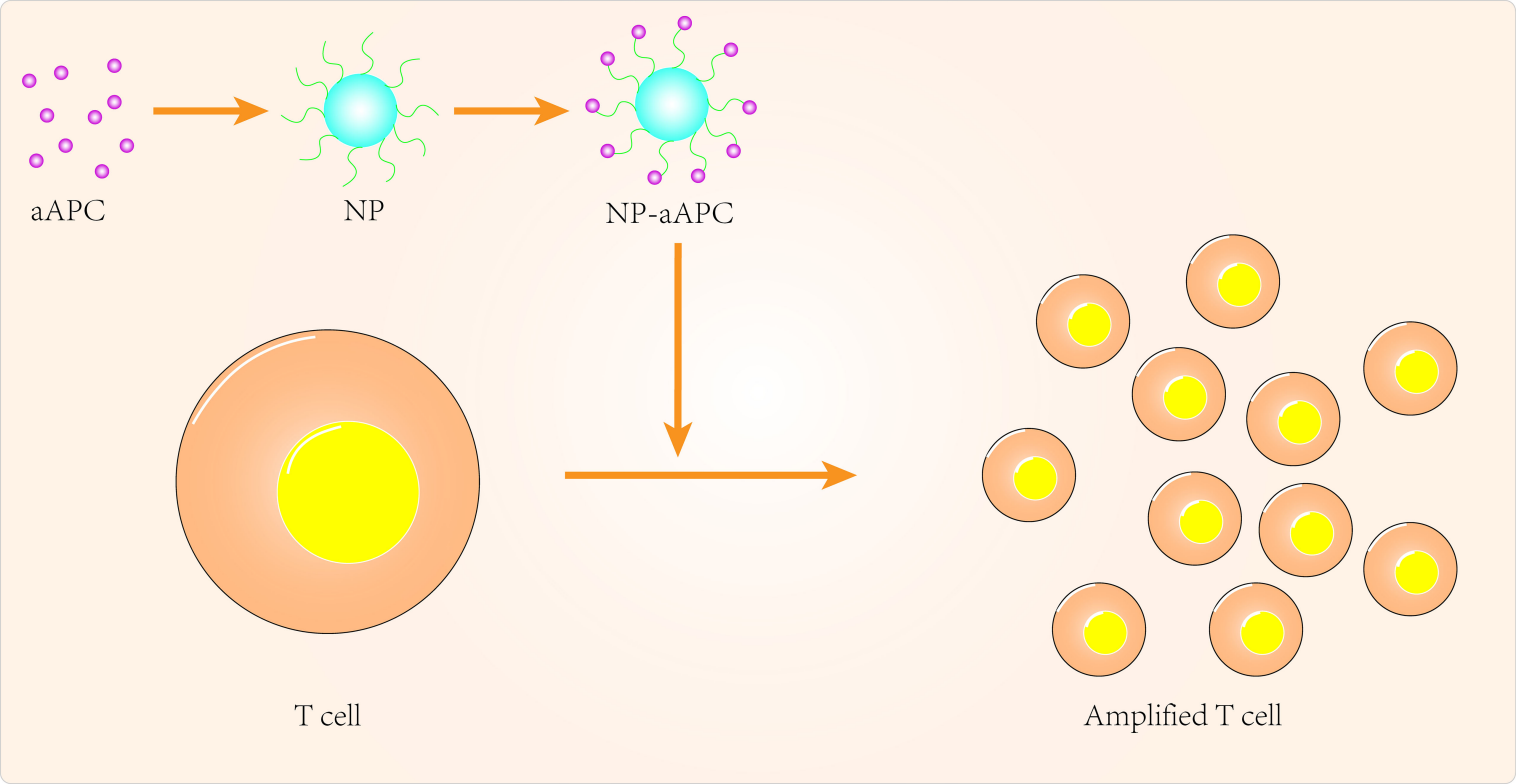
Targeted APCs to improve cancer immunotherapy.

Over the past few decades, tremendous progress has been made in the clinical application of peripatetic immunotherapy in the fight against tumors. However, given the cost and complexity of generating tumor-specific T cells, there are many limitations to the practical clinical application of secondary immunotherapy. Nanomedicine can provide additional technical support to overcome these limitations (**Figure 1**). Using artificial antigen-presenting cells (aAPCs), scientists have loaded specific MHC peptides and co-stimulatory molecules onto nanoparticles to activate and promote the expansion of antigen-specific CD8^+^ T cells (99). In some aAPCs, cytokines can also be used to promote activation and expansion of T cells in lymph nodes (100, 101). Iron-dextran nanoparticles and anti-biotin protein-coated quantum dot crystals have been synthesized using surface-coated MHC-I peptide complexes and biotin-CD28. These nanoparticles(NPs) are magnetic and can promote T-cell enrichment and activation using magnetic fields to promote TCR aggregation (102). IL-2 can activate T cells and with this in mind, Steenblock et al (100) constructed carbon nanotube polymers as aAPCs in which IL-2 was encapsulated and modified with MHC-I and αCD28. These aAPCs can induce activation and expansion of CD8^+^T cells with very low concentrations of IL-2 compared to conventional methods. In addition, Kelly et al (103) synthesized PLGA/PBAE aAPCs from poly(lactic acid-glycolic acid) (PLGA) and cationic polybasic amino esters (PBAE), nanoparticles that can expand antigen-specific cytotoxic CD8^+^ T cells *in vivo*.

As current T cell expansion rates are not ideal, research should also focus on biomaterials to improve *in vitro* T cell expansion (101, 102, 104). Cheung et al (105) designed an APC mimetic scaffold (APC-ms) that mimics natural APCs and compared this APC-ms to commercial expansion beads. This APC-ms promoted polyclonal expansion of mouse and human T cell generations by 2-10 times compared to commercial expansion beads (Dynabeads). The APC-ms consist of a liquid lipid bilayer supported by mesoporous silica microrods. After a single stimulation, the APC-ms resulted in antigen-specific expansion of a subpopulation of cytotoxic T cells in much larger numbers than autologous monocyte-derived dendritic cells. aAPCs may become the next focus of research in cancer nanomedicine, so more attention needs to be invested in optimizing the physicochemical properties of aAPCs, for example.

#### Targeting tumor cell-associated signaling pathway

Given the important role of PTEN in immunotherapy resistance, the design of PTEN-targeted nanomaterials has been the focus of many scientists. Kinoh et al (106) used pH-sensitive epirubicin-loaded micellar nanodrugs to synergize the efficacy of anti-PD-1 antibody (aPD-1) against PTEN-positive and PTEN-negative glioblastoma *in situ* (GBM). The combination of epirubicin-loaded micelles (Epi/m) with aPD-1 overcame the resistance of GBM to ICIs and reduced PD-L1 expression on tumor cells by inducing immunogenic cell death (ICD), eliminating MDSC and transforming the otherwise immune insensitive GBM into a hot tumor with high infiltration of anti-tumor immune cells. Catania et al (107) designed a combined local treatment based on Adriamycin (DOX) as an inducer of ICD and CpG (toll-like receptor-9 agonist, TLR-9 agonist) to synergistically eliminate GBM and found that a single intratumoral administration of HA-DOX + HA-CpG was effective in prolonging the survival of GBM animals. Teo et al (108) combined siRNA with folic acid-modified PEI, resulting in significant PD-1 silencing and enhanced T-cell activation. Yang et al (109) combined anti-PD-L1(aPD-L1) with multiple polyethylene glycol (PEG) chains to improve the efficacy and safety of checkpoint blockade treatment in GBM. In mice with GBM in situ, single doses of glycosylated and polyethylene glycol-linked antibodies reactivated anti-tumor immune responses and induced immune memory to protect the animals from recurrent tumor cell attack and to suppress autoimmune responses in healthy tissues of the animals.

**Figure 2:**
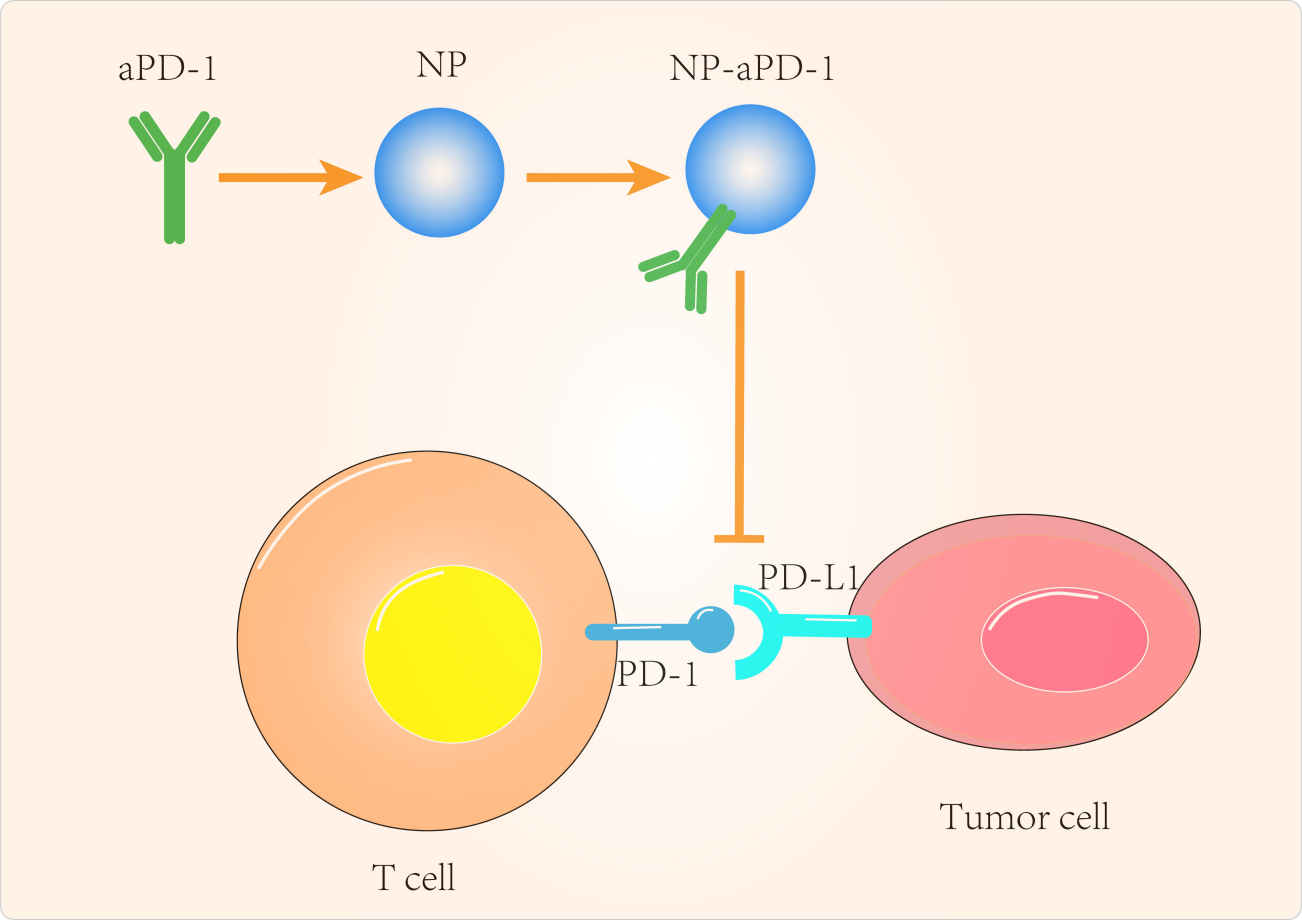
Targeted tumor cell-Associated signaling pathway to improve cancer immunotherapy.

In addition to GBM, PTEN-targeted nanomaterials have been investigated for other tumor applications. Lin et al (110) delivered mRNA *via* polymeric nanoparticles to effectively induce PTEN expression in melanoma and prostate cancer cells, which in turn induced autophagy and triggered cell death-related immune activation by releasing damage-related molecular patterns. *In vivo* experiments showed that PTEN mRNA nanoparticles reversed the immunosuppressive TME by promoting CD8^+^ T cell infiltration into tumor tissue, increasing pro-inflammatory cytokine expression and reducing Treg cells and MDSCs. The combination of PTEN mRNA nanoparticles with immune checkpoint inhibitors, aPD-1 antibodies, in a subcutaneous model of PTEN-mutant melanoma and PTEN-negative prostate cancer *in situ* models, produced potent anti-tumor effects (**Figure 2**).

### Microenvironment

#### Targeting immune checkpoint

When anti-CTLA4 antibodies are loaded into functionalized mesoporous silica (FMS) and administered intratumorally, they prolong local release under physiological conditions compared to free antibodies. FMS can be non-covalently linked to antibodies to promote sustained prolonged release (111, 112). Blockade of PD-1 with siRNA loaded into cationic lipids and polymeric NPs has been investigated (112). Wang et al (113) combined the synergistic delivery of anti-PD-1 antibodies and CpG oligodeoxynucleotides to prevent cancer recurrence. ye et al (114) demonstrated dual targeting of IDO and PD-1 by a microneedle-based transdermal delivery approach. Xiao et al (115) designed aPD-L1 functionalized mimetic polydopamine-modified gold nanostellar nanoparticles (PDA/GNS@aPD-L1 NPs) which, in addition to disrupting PD-1/PD-L1 immunosuppressive signaling, aPD-L1 scFv on the membranes of PDA/GNS@aPD-L1 NPs contributed to the accumulation of PDA-GNS at tumor sites. Importantly, PDA-GNS-induced photothermal ablation of tumors reverses the immunosuppressive tumor microenvironment, further enhancing the efficacy of PD-1/PD-L1 blockade therapy.

Combination immunotherapy with PD-L1 antibodies and CXCL12 inhibitors has better anti-tumor efficacy than single immunotherapy (116, 117). However, combination therapy has some drawbacks, including unpredictable PK/PD and overlapping toxicity. Therefore, targeting PD-L1 while reducing the duration of drug administration *in vivo* may help to reduce the cytotoxicity caused by free aPD-L1. Based on this concept, Miao et al (118) designed a chimeric PD-L1 trap protein. When a plasmid encoding the PD-L1 trap protein was encapsulated in lipid-fisetin DNA NPs and delivered to CT26 colon tumors and KPC pancreatic tumors, the highest expression was observed from day 2 to day 4 and finally declined by day 6. This transient expression and high affinity for PD-L1 molecules could serve as a promising therapeutic approach with reduced side effects.

#### Targeting TAMs

**Figure 3:**
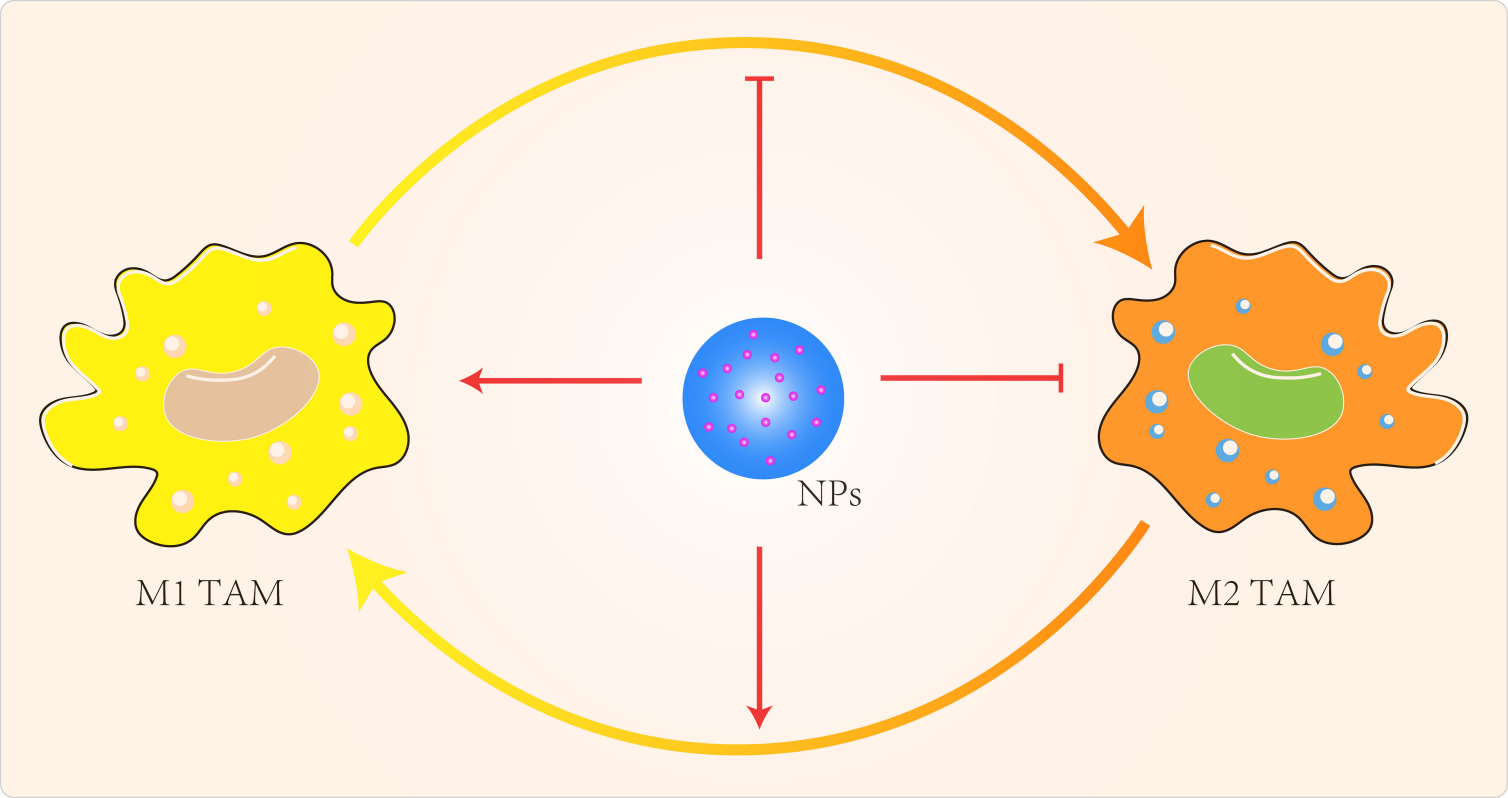
Targeted TAMs to improve cancer immunotherapy.

NP can also regulate TAMs by inhibiting macrophage recruitment, by inhibiting TAMs survival through the use of chemicals, by enhancing the activity of M1-type TAMs and by blocking the activity of M2-type TAMs (**Figure 3**) (119). Qian et al (120) used a novel bi-peptide in which scavenger receptor B type 1 (SRB1) was linked to a specific TAMs binding peptide which specifically blocked the M2-TAMs survival signals. Alternatively, we could try to reprogramme M2-type TAMs back to M1-type (121–123). When IL-12 in poly(β-amino ester) NPs was administered intravenously in a B16F10 melanoma model, M2-type macrophages were reduced and M1-type macrophages were increased (121). Specific miRNA-125b increased expression of MHCII, CD40, CD80, CD86 and increased responsiveness to IFN-γ in macrophages (124). Parayath et al (125) intraperitoneal injection of miRNA 125-b complexed with hyaluronic acid-fixed poly(ethyleneimine) NPs into the TME of G12/P53 mice resulted in increased M1-type macrophages and decreased M2-type macrophages. Zanganeh et al (126) repolarized M2-type TAMs back to M1-type *via* hydroxyl radicals for breast cancer treatment. Macrophages produce H2O2, which can be converted to cytotoxic hydroxyl radicals *via* iron oxide NPs. When co-cultures of macrophages and MMTV-PyMT cancer cells were treated with iron oxide NPs, there was an increase in the number of M1-type cells and a decrease in the number of M2-type cells. The effect of M1-type TAMs in the TME could be maximized by the use of immune checkpoint blockers or combination therapy with NPs carrying these modulators. Parayath et al (127) found that hyaluronic acid-based nanoparticles encapsulated with miR-125b (HA-PEI-miR-125b) could specifically target homozygous ID8-VEGF ovarian cancer mouse The imbalance in the ratio of M1 to M2 TAMs populations and the uncontrolled increase of M1 TAMs stimulate the inflammatory response. In contrast, TAMs can sometimes express both M1- and M2-type markers (128), which limits the application of specific targeting of M2-type TAMs.

#### Targeting MDSCs

Strategies to regulate MDSCs primarily require specific blockade of MDSCs development, differentiation of MDSCs into mature cells, and depletion and inhibition of MDSC function by small molecule drugs (129–133). Gold NPs based on high-density lipoprotein (HDL) loaded with apoA-1 have been used to inhibit MDSCs, and in a study in B16F10 melanoma mice, MDSCs were depleted by lipid-encapsulated calcium phosphate NPs loaded with gemcitabine (134, 135). Plebanek et al (136) synthesized a HDL receptor with high affinity for HDL that inhibited MDSCs activity by specifically binding SCARB1. In another study, Kong et al (137) used mesoporous silica NPs loaded with all-trans retinoic acid and Dox, coated with IL-2 and subsequently modified with dipalmitoylphosphatidylcholine cholesterol and DSPE-PEG 2000. Intravenous administration of these NPs reduced the MDSCs population by 2.7-fold and increased the population of NK cells, mature DCs and cytotoxic T cells in the TME. Similarly, IL-2 encapsulated NPs were designed to remodel the TME cell population, but were associated with poor secondary cytokine responses (138).

#### Targeting CAFs

Blocking the pathway activated by CAFs can reverse mesenchymal-mediated multidrug resistance. In one study, targeting CAFs with anti-FAP SCFV-modified ferritin nanomaterials containing the photosensitizer zinc hexadecafluorophthalocyanine led to CAF ablation and increased the number of CD8^+^ T cells in the TME when irradiated with 671nm light (139). Intraperitoneal injection of homogeneous gold nanoparticles in the ASPC1 human pancreatic cancer cell and CAF19 pancreatic stellate tumor mouse models led to a reduction in fibronectin, collagen and α-SMA (140). These NPs specifically target α-SMA-positive CAFs through the interaction of serum albumin encapsulated on NPs during circulation with cysteine-rich acidic protein (SPARC) secreted on the surface of CAFs (141).

#### Targeting NK cells

NK cells play a pivotal role in tumor immunotherapy, and more and more scholars have started to design nanomedicines for NK cell-related immune resistance. Liu et al (142) designed a nanoemulsion system (SSB-NMs) to load TGF-β inhibitors and selenocysteine (Se-C), and the experimental results showed that the nanoparticles significantly enhanced the efficacy of NK cell-mediated immunotherapy against triple-negative breast cancer. Lai et al. (143) used a selenium-containing ruthenium complex (Ru-Se) to synergistically enhance NK cell-mediated killing of prostate cancer cells. The complex was found to effectively enhance NK cell lysis of PC3 cells and was demonstrated in 10 clinical patients. NK cells are superior to T cells in their ability to fight tumors in the absence of specific antigens, making them a potential target for tumor immunotherapy.

#### Targeting immune-related cytokines

Zaretsky et al (38) melanoma patients resistant to PD-1 treatment acquired loss-of-function mutations in JAK1/2. Although tumor cells were still recognized by T cells, their JAK1/2 mutation rendered them insensitive to the anti-proliferative effects of IFN-γ and they lacked IFN-γ induced PD-L1 and MHC class I surface expression. Similarly, analysis of tumors from patients resistant to treatment with the anti-CTLA4 drug Ipilimumab showed that mutations in the IFN-γ pathway genes IFNGR1/2, JAK1/2 and IRF1 inhibit the response of tumor cells to IFN-γ signaling (39). This facilitates tumor escape from T-cell immunity, thereby conferring resistance to anti-CTLA4 therapy.

After treatment with Frax NEs, the T cofactor 1 (Th1) cytokine of IFN-γ was effective in inducing anti-tumor immunity (144). Transforming growth factor-β (TGF-β), chemokine (C-C motif) ligand 2 (CCL2) and interleukin 6 (IL6), which inhibit the development of anti-tumor immunity, were reduced. Although Frax NEs have shown an inhibitory effect on tumor growth, this monotherapy is only partially effective against tumors and does not maintain the tumor growth effect long after administration has stopped.

#### Targeting Tregs

Lin et al. (110) constructed PTEN mRNA nanoparticles (mPTEN @ NPs) delivered to tumour cells with PTEN deletion or mutation to restore the TME, stimulate immune response and enhance the efficacy of immune checkpoint blockade (ICB) therapy by inducing autophagy activation and damage-associated molecular patterns (DAMPs) release. In their study, mPTEN @ NPs restored tumour sensitivity to immunotherapy and triggered the release of DAMPs and autophagy, thereby promoting the formation of autophagosomes. *In vivo* studies showed that PTEN repair induced a strong CD8^+^ T cell response and restored TME by inhibiting the production of Tregs and monocyte MDSCs and promoting the production of pro-inflammatory cytokines. In addition, they evaluated the anti-tumour effects of mPTEN @ NPs in combination with anti-PD-1 immunotherapy in a PTEN-deficient or mutated tumour model, showing that this combination therapy strategy has significant therapeutic efficacy and immunological memory. These results suggest that nanomedicine mRNA repair of tumour suppressors can enhance the sensitivity of tumors to ICB therapy and provide an effective cooperative treatment strategy for a variety of malignancies.

## Conclusion and perspective

Despite the remarkable achievements of immunotherapy in cancer treatment, all current treatment strategies have serious limitations and often encounter difficulties in clinical treatment. There are many reasons for treatment failure, including poor oral bioavailability of some drugs and serious drug-related side effects. The most challenging problem is that patients receiving immunotherapy almost always develop drug resistance. While much progress has been made in recent years in understanding the mechanisms of resistance to cancer immunotherapy, overcoming drug resistance remains an important unmet clinical need. Thus, immune resistance in tumors has become a major obstacle to cancer immunotherapy, and overcoming resistance is a goal actively pursued by current and future oncologists (145).

The emergence of nanotechnology offers a novel solution to immunotherapy resistance. Nanomedicines can improve the bioavailability of insoluble drugs, prolong drug circulation, cross the biological barrier, achieve tumor-targeted therapy through passive or active targeting, improve anti-tumor effects and reduce tumor resistance to immunotherapy. A number of nanomaterials have now completed Phase III clinical trials (**Table 1**).The convergence of these two disciplines will certainly provide a tremendous impetus to improve cancer treatment. The advantages of nanomedicines over current therapeutic strategies will continue to be exploited as more nanomedicines are developed and optimized. It is therefore believed that nanomedicines will be an attractive strategy for reversing or overcoming resistance to cancer immunotherapy.

**Table 1:** Summary of Phase 3 Clinical Trials for Cancer Nanomedicine Products.

Composition	Responsible Party	Indication	NCT number	Status
Liposomal paclitaxel	SynCore Biotechnology Co., Ltd.	Pancreatic adenocarcinoma	NCT03126435	Completed
Thermally sensitive liposomal doxorubicin	Imunon	Hepatocellular carcinoma	NCT00617981	Completed
Liposomal VCL-1005 plasmid	Vical	Melanoma	NCT00395070	Completed
Liposomal MUC1 antigen	EMD Serono	Non-small-cell lung cancer	NCT00409188	Completed
Hafnium-oxide nanoparticle	Nanobiotix	Sarcoma	NCT02379845	Completed
Hafnium-oxide nanoparticle	American Regent, Inc.	Non-small-cell lung cancer	NCT00243867	Completed
Irinotecan PEG conjugate	Nektar Therapeutics	Breast cancer	NCT02915744	Completed
			NCT01492101	Completed
Cisplatin micellar	Orient Europharma Co., Ltd.	Pancreatic cancer	NCT02043288	Completed
Paclitaxel micellar	Nippon Kayaku Co., Ltd.	Breast cancer	NCT01644890	Completed

Drug resistance in tumor immunotherapy involves multiple mechanisms working together, so here we have only reviewed the more mature aspects of nanomaterial development, as for other aspects of drug resistance mechanisms such as hypoxic environment (146, 147), lactate metabolism (148), glycolytic metabolism (149), tryptophan metabolism (150), cholesterol metabolism (151–154), which also play an important role in drug resistance. These mechanisms of drug resistance may also provide additional clues for the application of nanomedicine.

Currently, a lot of researchers are concentrating on creating organically produced NPs, which are safer as well as more biocompatible and able to activate the body’s own immune cells and use that immune response to help eradicate cancers (155). But in this case, there is a more subtle biological element and a well-defined treatment process. In conclusion, it is encouraging to see how NPs might increase the body’s immunological resilience. It is thought that through in-depth research in this area and the design of more effective nanomaterials, it is possible to overcome immune resistance, reduce toxic side effects on patients, and achieve better therapeutic effects for the benefit of cancer patients, despite some limitations in its clinical application.

## Author contributions

JS carried out the primary literature search, drafted and revised the manuscript. YJ helped modify the manuscript and participated in discussions. CJ conceived and approved the final manuscript. All authors contributed to the article and approved the submitted version.

## References

[1] Breakthrough of the year 2013. Notable developments. Science (2013) 342:1435–41. doi: 10.1126/science.342.6165.1444 24357296

[2] Vesely MD Zhang T Chen L. Resistance mechanisms to anti-PD cancer immunotherapy. Annu Rev Immunol (2022) 40:45–74. doi: 10.1146/annurev-immunol-070621-030155 35471840

[3] Jerby-Arnon L Shah P Cuoco MS Rodman C Su M-J Melms JC. A cancer cell program promotes T cell exclusion and resistance to checkpoint blockade. Cell (2018) 175:984–997.e24. doi: 10.1016/j.cell.2018.09.006 30388455 PMC6410377

[4] Baldominos P Barbera-Mourelle A Barreiro O Huang Y Wight A Cho J-W. Quiescent cancer cells resist T cell attack by forming an immunosuppressive niche. Cell (2022) 185:1694–1708.e19. doi: 10.1016/j.cell.2022.03.033 35447074 PMC11332067

[5] Yu J Green MD Li S Sun Y Journey SN Choi JE. Liver metastasis restrains immunotherapy efficacy *via* macrophage-mediated T cell elimination. Nat Med (2021) 27:152–64. doi: 10.1038/s41591-020-1131-x PMC8095049 33398162

[6] Goc J Lv M Bessman NJ Flamar A-L Sahota S Suzuki H. Dysregulation of ILC3s unleashes progression and immunotherapy resistance in colon cancer. Cell (2021) 184:5015–5030.e16. doi: 10.1016/j.cell.2021.07.029 34407392 PMC8454863

[7] Wu P Han J Gong Y Liu C Yu H Xie N. Nanoparticle-based drug delivery systems targeting tumor microenvironment for cancer immunotherapy resistance: current advances and applications. Pharmaceutics (2022) 14:1990. doi: 10.3390/pharmaceutics14101990 36297426 PMC9612242

[8] Shen L Li J Liu Q Song W Zhang X Tiruthani K. Local blockade of interleukin 10 and C-X-C motif chemokine ligand 12 with nano-delivery promotes antitumor response in murine cancers. ACS Nano (2018) 12:9830–41. doi: 10.1021/acsnano.8b00967 30253648

[9] Gao S Yang D Fang Y Lin X Jin X Wang Q. Engineering nanoparticles for targeted remodeling of the tumor microenvironment to improve cancer immunotherapy. Theranostics (2019) 9:126–51. doi: 10.7150/thno.29431 PMC6332787 30662558

[10] Chen Y. Nanotechnology for next-generation cancer immunotherapy: State of the art and future perspectives. J Control Release (2023) 356:14–25. doi: 10.1016/j.jconrel.2023.02.016 36805873

[11] Riley RS June CH Langer R Mitchell MJ. Delivery technologies for cancer immunotherapy. Nat Rev Drug Discovery (2019) 18:175–96. doi: 10.1038/s41573-018-0006-z PMC6410566 30622344

[12] Xu Y Xiong J Sun X Gao H. Targeted nanomedicines remodeling immunosuppressive tumor microenvironment for enhanced cancer immunotherapy. Acta Pharm Sin B (2022) 12:4327–47. doi: 10.1016/j.apsb.2022.11.001 PMC9764075 36561994

[13] Reeves E James E. Antigen processing and immune regulation in the response to tumours. Immunology (2017) 150:16–24. doi: 10.1111/imm.12675 27658710 PMC5341504

[14] Łuksza M Riaz N Makarov V Balachandran VP Hellmann MD Solovyov A. A neoantigen fitness model predicts tumour response to checkpoint blockade immunotherapy. Nature (2017) 551:517–20. doi: 10.1038/nature24473 PMC6137806 29132144

[15] Carbone DP Reck M Paz-Ares L Creelan B Horn L Steins M. First-line nivolumab in stage IV or recurrent non-small-cell lung cancer. N Engl J Med (2017) 376:2415–26. doi: 10.1056/NEJMoa1613493 PMC6487310 28636851

[16] Schumacher TN Schreiber RD. Neoantigens in cancer immunotherapy. Science (2015) 348:69–74. doi: 10.1126/science.aaa4971 25838375

[17] Le DT Uram JN Wang H Bartlett BR Kemberling H Eyring AD. PD-1 blockade in tumors with mismatch-repair deficiency. N Engl J Med (2015) 372:2509–20. doi: 10.1056/NEJMoa1500596 PMC4481136 26028255

[18] Le DT Durham JN Smith KN Wang H Bartlett BR Aulakh LK. Mismatch repair deficiency predicts response of solid tumors to PD-1 blockade. Science (2017) 357:409–13. doi: 10.1126/science.aan6733 PMC5576142 28596308

[19] Lawrence MS Stojanov P Polak P Kryukov GV Cibulskis K Sivachenko A. Mutational heterogeneity in cancer and the search for new cancer-associated genes. Nature (2013) 499:214–8. doi: 10.1038/nature12213 PMC3919509 23770567

[20] de Vries TJ Fourkour A Wobbes T Verkroost G Ruiter DJ van Muijen GN. Heterogeneous expression of immunotherapy candidate proteins gp100, MART-1, and tyrosinase in human melanoma cell lines and in human melanocytic lesions. Cancer Res (1997) 57:3223–9. 9242453

[21] DuPage M Mazumdar C Schmidt LM Cheung AF Jacks T. Expression of tumour-specific antigens underlies cancer immunoediting. Nature (2012) 482:405–9. doi: 10.1038/nature10803 PMC3288744 22318517

[22] Bai X-F Liu J Li O Zheng P Liu Y. Antigenic drift as a mechanism for tumor evasion of destruction by cytolytic T lymphocytes. J Clin Invest (2003) 111:1487–96. doi: 10.1172/JCI17656 PMC155049 12750398

[23] Galluzzi L Buqué A Kepp O Zitvogel L Kroemer G. Immunological effects of conventional chemotherapy and targeted anticancer agents. Cancer Cell (2015) 28:690–714. doi: 10.1016/j.ccell.2015.10.012 26678337

[24] Kroemer G Galluzzi L Kepp O Zitvogel L. Immunogenic cell death in cancer therapy. Annu Rev Immunol (2013) 31:51–72. doi: 10.1146/annurev-immunol-032712-100008 23157435

[25] Fucikova J Becht E Iribarren K Goc J Remark R Damotte D. Calreticulin expression in human non-small cell lung cancers correlates with increased accumulation of antitumor immune cells and favorable prognosis. Cancer Res (2016) 76:1746–56. doi: 10.1158/0008-5472.CAN-15-1142 26842877

[26] Ladoire S Penault-Llorca F Senovilla L Dalban C Enot D Locher C. Combined evaluation of LC3B puncta and HMGB1 expression predicts residual risk of relapse after adjuvant chemotherapy in breast cancer. Autophagy (2015) 11:1878–90. doi: 10.1080/15548627.2015.1082022 PMC4824597 26506894

[27] Peng W Chen JQ Liu C Malu S Creasy C Tetzlaff MT. Loss of PTEN promotes resistance to T cell-mediated immunotherapy. Cancer Discovery (2016) 6:202–16. doi: 10.1158/2159-8290.CD-15-0283 PMC4744499 26645196

[28] Cretella D Digiacomo G Giovannetti E Cavazzoni A. PTEN alterations as a potential mechanism for tumor cell escape from PD-1/PD-L1 inhibition. Cancers (Basel) (2019) 11:1318. doi: 10.3390/cancers11091318 31500143 PMC6770107

[29] Cetintas VB Batada NN. Is there a causal link between PTEN deficient tumors and immunosuppressive tumor microenvironment? J Transl Med (2020) 18:45. doi: 10.1186/s12967-020-02219-w 32000794 PMC6993356

[30] Feng S Cheng X Zhang L Lu X Chaudhary S Teng R. Myeloid-derived suppressor cells inhibit T cell activation through nitrating LCK in mouse cancers. Proc Natl Acad Sci U.S.A. (2018) 115:10094–9. doi: 10.1073/pnas.1800695115 PMC6176562 30232256

[31] Sharma MD Shinde R McGaha TL Huang L Holmgaard RB Wolchok JD. The PTEN pathway in Tregs is a critical driver of the suppressive tumor microenvironment. Sci Adv (2015) 1:e1500845. doi: 10.1126/sciadv.1500845 26601142 PMC4640592

[32] Piro G Carbone C Carbognin L Pilotto S Ciccarese C Iacovelli R. Revising PTEN in the era of immunotherapy: new perspectives for an old story. Cancers (Basel) (2019) 11:1525. doi: 10.3390/cancers11101525 31658667 PMC6826982

[33] Vidotto T Melo CM Castelli E Koti M Dos Reis RB Squire JA. Emerging role of PTEN loss in evasion of the immune response to tumours. Br J Cancer (2020) 122:1732–43. doi: 10.1038/s41416-020-0834-6 PMC7283470 32327707

[34] Conciatori F Bazzichetto C Falcone I Ciuffreda L Ferretti G Vari S. PTEN Function at the Interface between Cancer and Tumor Microenvironment: Implications for Response to Immunotherapy. Int J Mol Sci (2020) 21:5337. doi: 10.3390/ijms21155337 32727102 PMC7432882

[35] Arico S Petiot A Bauvy C Dubbelhuis PF Meijer AJ Codogno P. The tumor suppressor PTEN positively regulates macroautophagy by inhibiting the phosphatidylinositol 3-kinase/protein kinase B pathway. J Biol Chem (2001) 276:35243–6. doi: 10.1074/jbc.C100319200 11477064

[36] Darnell JE Kerr IM Stark GR. Jak-STAT pathways and transcriptional activation in response to IFNs and other extracellular signaling proteins. Science (1994) 264:1415–21. doi: 10.1126/science.8197455 8197455

[37] Manguso RT Pope HW Zimmer MD Brown FD Yates KB Miller BC. *In vivo* CRISPR screening identifies Ptpn2 as a cancer immunotherapy target. Nature (2017) 547:413–8. doi: 10.1038/nature23270 PMC5924693 28723893

[38] Zaretsky JM Garcia-Diaz A Shin DS Escuin-Ordinas H Hugo W Hu-Lieskovan S. Mutations associated with acquired resistance to PD-1 blockade in melanoma. N Engl J Med (2016) 375:819–29. doi: 10.1056/NEJMoa1604958 PMC5007206 27433843

[39] Possick JD. Pulmonary toxicities from checkpoint immunotherapy for Malignancy. Clin Chest Med (2017) 38:223–32. doi: 10.1016/j.ccm.2016.12.012 28477635

[40] Robert C Schachter J Long GV Arance A Grob JJ Mortier L. Pembrolizumab versus ipilimumab in advanced melanoma. N Engl J Med (2015) 372:2521–32. doi: 10.1056/NEJMoa1503093 25891173

[41] Kandel S Adhikary P Li G Cheng K. The TIM3/Gal9 signaling pathway: An emerging target for cancer immunotherapy. Cancer Lett (2021) 510:67–78. doi: 10.1016/j.canlet.2021.04.011 33895262 PMC8168453

[42] Pathria P Louis TL Varner JA. Targeting tumor-associated macrophages in cancer. Trends Immunol (2019) 40:310–27. doi: 10.1016/j.it.2019.02.003 30890304

[43] Xiang X Wang J Lu D Xu X. Targeting tumor-associated macrophages to synergize tumor immunotherapy. Signal Transduct Target Ther (2021) 6:75. doi: 10.1038/s41392-021-00484-9 33619259 PMC7900181

[44] Chanmee T Ontong P Konno K Itano N. Tumor-associated macrophages as major players in the tumor microenvironment. Cancers (Basel) (2014) 6:1670–90. doi: 10.3390/cancers6031670 PMC4190561 25125485

[45] Noy R Pollard JW. Tumor-associated macrophages: from mechanisms to therapy. Immunity (2014) 41:49–61. doi: 10.1016/j.immuni.2014.06.010 25035953 PMC4137410

[46] Ramanathan S Jagannathan N. Tumor associated macrophage: a review on the phenotypes, traits and functions. Iran J Cancer Prev (2014) 7:1–8. 25250141 PMC4142950

[47] Lin Y Xu J Lan H. Tumor-associated macrophages in tumor metastasis: biological roles and clinical therapeutic applications. J Hematol Oncol (2019) 12:76. doi: 10.1186/s13045-019-0760-3 31300030 PMC6626377

[48] Neubert NJ Schmittnaegel M Bordry N Nassiri S Wald N Martignier C. T cell-induced CSF1 promotes melanoma resistance to PD1 blockade. Sci Transl Med (2018) 10:eaan3311. doi: 10.1126/scitranslmed.aan3311 29643229 PMC5957531

[49] Mok S Koya RC Tsui C Xu J Robert L Wu L. Inhibition of CSF-1 receptor improves the antitumor efficacy of adoptive cell transfer immunotherapy. Cancer Res (2014) 74:153–61. doi: 10.1158/0008-5472.CAN-13-1816 PMC3947337 24247719

[50] Zhu Y Yang J Xu D Gao X-M Zhang Z Hsu JL. Disruption of tumour-associated macrophage trafficking by the osteopontin-induced colony-stimulating factor-1 signalling sensitises hepatocellular carcinoma to anti-PD-L1 blockade. Gut (2019) 68:1653–66. doi: 10.1136/gutjnl-2019-318419 30902885

[51] Zhu Y Knolhoff BL Meyer MA Nywening TM West BL Luo J. CSF1/CSF1R blockade reprograms tumor-infiltrating macrophages and improves response to T-cell checkpoint immunotherapy in pancreatic cancer models. Cancer Res (2014) 74:5057–69. doi: 10.1158/0008-5472.CAN-13-3723 PMC4182950 25082815

[52] Hu W Li X Zhang C Yang Y Jiang J Wu C. Tumor-associated macrophages in cancers. Clin Transl Oncol (2016) 18:251–8. doi: 10.1007/s12094-015-1373-0 26264497

[53] Jung KY Cho SW Kim YA Kim D Oh B-C Park DJ. Cancers with higher density of tumor-associated macrophages were associated with poor survival rates. J Pathol Transl Med (2015) 49:318–24. doi: 10.4132/jptm.2015.06.01 PMC4508569 26081823

[54] Fritz JM Tennis MA Orlicky DJ Lin H Ju C Redente EF. Depletion of tumor-associated macrophages slows the growth of chemically induced mouse lung adenocarcinomas. Front Immunol (2014) 5:587. doi: 10.3389/fimmu.2014.00587 25505466 PMC4243558

[55] Wu X Schulte BC Zhou Y Haribhai D Mackinnon AC Plaza JA. Depletion of M2-like tumor-associated macrophages delays cutaneous T-cell lymphoma development in *vivo* . J Invest Dermatol (2014) 134:2814–22. doi: 10.1038/jid.2014.206 24780929

[56] Ries CH Cannarile MA Hoves S Benz J Wartha K Runza V. Targeting tumor-associated macrophages with anti-CSF-1R antibody reveals a strategy for cancer therapy. Cancer Cell (2014) 25:846–59. doi: 10.1016/j.ccr.2014.05.016 24898549

[57] Luo Y Zhou H Krueger J Kaplan C Lee S-H Dolman C. Targeting tumor-associated macrophages as a novel strategy against breast cancer. J Clin Invest (2006) 116:2132–41. doi: 10.1172/JCI27648 PMC1513049 16862213

[58] Ruffell B Chang-Strachan D Chan V Rosenbusch A Ho CMT Pryer N. Macrophage IL-10 blocks CD8+ T cell-dependent responses to chemotherapy by suppressing IL-12 expression in intratumoral dendritic cells. Cancer Cell (2014) 26:623–37. doi: 10.1016/j.ccell.2014.09.006 PMC4254570 25446896

[59] Veglia F Sanseviero E Gabrilovich DI. Myeloid-derived suppressor cells in the era of increasing myeloid cell diversity. Nat Rev Immunol (2021) 21:485–98. doi: 10.1038/s41577-020-00490-y PMC7849958 33526920

[60] Solito S Falisi E Diaz-Montero CM Doni A Pinton L Rosato A. A human promyelocytic-like population is responsible for the immune suppression mediated by myeloid-derived suppressor cells. Blood (2011) 118:2254–65. doi: 10.1182/blood-2010-12-325753 PMC3709641 21734236

[61] Si Y Merz SF Jansen P Wang B Bruderek K Altenhoff P. Multidimensional imaging provides evidence for down-regulation of T cell effector function by MDSC in human cancer tissue. Sci Immunol (2019) 4:eaaw9159. doi: 10.1126/sciimmunol.aaw9159 31628161

[62] Kodumudi KN Weber A Sarnaik AA Pilon-Thomas S. Blockade of myeloid-derived suppressor cells after induction of lymphopenia improves adoptive T cell therapy in a murine model of melanoma. J Immunol (2012) 189:5147–54. doi: 10.4049/jimmunol.1200274 PMC3505990 23100512

[63] Meyer C Cagnon L Costa-Nunes CM Baumgaertner P Montandon N Leyvraz L. Frequencies of circulating MDSC correlate with clinical outcome of melanoma patients treated with ipilimumab. Cancer Immunol Immunother (2014) 63:247–57. doi: 10.1007/s00262-013-1508-5 PMC11029062 24357148

[64] Yang L DeBusk LM Fukuda K Fingleton B Green-Jarvis B Shyr Y. Expansion of myeloid immune suppressor Gr+CD11b+ cells in tumor-bearing host directly promotes tumor angiogenesis. Cancer Cell (2004) 6:409–21. doi: 10.1016/j.ccr.2004.08.031 15488763

[65] Yang L Huang J Ren X Gorska AE Chytil A Aakre M. Abrogation of TGF beta signaling in mammary carcinomas recruits Gr-1+CD11b+ myeloid cells that promote metastasis. Cancer Cell (2008) 13:23–35. doi: 10.1016/j.ccr.2007.12.004 18167337 PMC2245859

[66] Li K Shi H Zhang B Ou X Ma Q Chen Y. Myeloid-derived suppressor cells as immunosuppressive regulators and therapeutic targets in cancer. Signal Transduct Target Ther (2021) 6:362. doi: 10.1038/s41392-021-00670-9 34620838 PMC8497485

[67] Fujimura T Kambayashi Y Aiba S. Crosstalk between regulatory T cells (Tregs) and myeloid derived suppressor cells (MDSCs) during melanoma growth. Oncoimmunology (2012) 1:1433–4. doi: 10.4161/onci.21176 PMC3518528 23243619

[68] Wesolowski R Markowitz J Carson WE. Myeloid derived suppressor cells - a new therapeutic target in the treatment of cancer. J Immunother Cancer (2013) 1:10. doi: 10.1186/2051-1426-1-10 24829747 PMC4019895

[69] Sahai E Astsaturov I Cukierman E DeNardo DG Egeblad M Evans RM. A framework for advancing our understanding of cancer-associated fibroblasts. Nat Rev Cancer (2020) 20:174–86. doi: 10.1038/s41568-019-0238-1 PMC7046529 31980749

[70] Servais C Erez N. From sentinel cells to inflammatory culprits: cancer-associated fibroblasts in tumour-related inflammation. J Pathol (2013) 229:198–207. doi: 10.1002/path.4103 22996812

[71] Deng Y Cheng J Fu B Liu W Chen G Zhang Q. Hepatic carcinoma-associated fibroblasts enhance immune suppression by facilitating the generation of myeloid-derived suppressor cells. Oncogene (2017) 36:1090–101. doi: 10.1038/onc.2016.273 27593937

[72] Takahashi H Sakakura K Kudo T Toyoda M Kaira K Oyama T. Cancer-associated fibroblasts promote an immunosuppressive microenvironment through the induction and accumulation of protumoral macrophages. Oncotarget (2017) 8:8633–47. doi: 10.18632/oncotarget.14374 PMC5352428 28052009

[73] Komohara Y Takeya M. CAFs and TAMs: maestros of the tumour microenvironment. J Pathol (2017) 241:313–5. doi: 10.1002/path.4824 27753093

[74] Cheng J-T Deng Y-N Yi H-M Wang G-Y Fu B-S Chen W-J. Hepatic carcinoma-associated fibroblasts induce IDO-producing regulatory dendritic cells through IL-6-mediated STAT3 activation. Oncogenesis (2016) 5:e198. doi: 10.1038/oncsis.2016.7 26900950 PMC5154347

[75] Pinchuk IV Saada JI Beswick EJ Boya G Qiu SM Mifflin RC. PD-1 ligand expression by human colonic myofibroblasts/fibroblasts regulates CD4+ T-cell activity. Gastroenterology (2008) 135:1228–1237, 1237.e1–2. doi: 10.1053/j.gastro.2008.07.016 18760278 PMC2584612

[76] Ghebeh H Dermime S. Comment on “Characterization of human lung tumor-associated fibroblasts and their ability to modulate the activation of tumor-associated T cells”. J Immunol (2007) 179:732. doi: 10.4049/jimmunol.179.2.732 17617559

[77] Chiossone L Dumas P-Y Vienne M Vivier E. Natural killer cells and other innate lymphoid cells in cancer. Nat Rev Immunol (2018) 18:671–88. doi: 10.1038/s41577-018-0061-z 30209347

[78] Cifaldi L Di Santo J Olive D. Editorial: molecular strategies aimed to boost NK cell-based immunotherapy of cancer. Front Immunol (2020) 11:1132. doi: 10.3389/fimmu.2020.01132 32612604 PMC7308425

[79] Tarannum M Romee R Shapiro RM. Innovative strategies to improve the clinical application of NK cell-based immunotherapy. Front Immunol (2022) 13:859177. doi: 10.3389/fimmu.2022.859177 35401529 PMC8990319

[80] Guillerey C Huntington ND Smyth MJ. Targeting natural killer cells in cancer immunotherapy. Nat Immunol (2016) 17:1025–36. doi: 10.1038/ni.3518 27540992

[81] Mikelez-Alonso I Magadán S González-Fernández Á Borrego F. Natural killer (NK) cell-based immunotherapies and the many faces of NK cell memory: A look into how nanoparticles enhance NK cell activity. Adv Drug Delivery Rev (2021) 176:113860. doi: 10.1016/j.addr.2021.113860 34237404

[82] Otegbeye F Ojo E Moreton S Mackowski N Lee DA de Lima M. Inhibiting TGF-beta signaling preserves the function of highly activated, in *vitro* expanded natural killer cells in AML and colon cancer models. PloS One (2018) 13:e0191358. doi: 10.1371/journal.pone.0191358 29342200 PMC5771627

[83] Regis S Dondero A Caliendo F Bottino C Castriconi R. NK cell function regulation by TGF-β-induced epigenetic mechanisms. Front Immunol (2020) 11:311. doi: 10.3389/fimmu.2020.00311 32161594 PMC7052483

[84] Huang C-H Liao Y-J Chiou T-J Huang H-T Lin Y-H Twu Y-C. TGF-β regulated leukemia cell susceptibility against NK targeting through the down-regulation of the CD48 expression. Immunobiology (2019) 224:649–58. doi: 10.1016/j.imbio.2019.07.002 31421859

[85] Shi L Lin H Li G Sun Y Shen J Xu J. Cisplatin enhances NK cells immunotherapy efficacy to suppress HCC progression *via* altering the androgen receptor (AR)-ULBP2 signals. Cancer Lett (2016) 373:45–56. doi: 10.1016/j.canlet.2016.01.017 26805759 PMC4887098

[86] Yoo JY Jaime-Ramirez AC Bolyard C Dai H Nallanagulagari T Wojton J. Bortezomib treatment sensitizes oncolytic HSV-1-treated tumors to NK cell immunotherapy. Clin Cancer Res (2016) 22:5265–76. doi: 10.1158/1078-0432.CCR-16-1003 PMC5093037 27390350

[87] Padrón LJ Maurer DM O’Hara MH O’Reilly EM Wolff RA Wainberg ZA. Sotigalimab and/or nivolumab with chemotherapy in first-line metastatic pancreatic cancer: clinical and immunologic analyses from the randomized phase 2 PRINCE trial. Nat Med (2022) 28:1167–77. doi: 10.1038/s41591-022-01829-9 PMC9205784 35662283

[88] Platanias LC. Mechanisms of type-I- and type-II-interferon-mediated signalling. Nat Rev Immunol (2005) 5:375–86. doi: 10.1038/nri1604 15864272

[89] Gao J Shi LZ Zhao H Chen J Xiong L He Q. Loss of IFN-γ Pathway genes in tumor cells as a mechanism of resistance to anti-CTLA-4 therapy. Cell (2016) 167:397–404.e9. doi: 10.1016/j.cell.2016.08.069 27667683 PMC5088716

[90] Ohue Y Nishikawa H. Regulatory T (Treg) cells in cancer: Can Treg cells be a new therapeutic target? Cancer Sci (2019) 110:2080–9. doi: 10.1111/cas.14069 PMC6609813 31102428

[91] Sundstedt A O’Neill EJ Nicolson KS Wraith DC. Role for IL-10 in suppression mediated by peptide-induced regulatory T cells in *vivo* . J Immunol (2003) 170:1240–8. doi: 10.4049/jimmunol.170.3.1240 12538682

[92] Sakaguchi S Yamaguchi T Nomura T Ono M. Regulatory T cells and immune tolerance. Cell (2008) 133:775–87. doi: 10.1016/j.cell.2008.05.009 18510923

[93] Oida T Zhang X Goto M Hachimura S Totsuka M Kaminogawa S. CD4+CD25- T cells that express latency-associated peptide on the surface suppress CD4+CD45RBhigh-induced colitis by a TGF-beta-dependent mechanism. J Immunol (2003) 170:2516–22. doi: 10.4049/jimmunol.170.5.2516 12594277

[94] Nishikawa H Koyama S. Mechanisms of regulatory T cell infiltration in tumors: implications for innovative immune precision therapies. J Immunother Cancer (2021) 9:e002591. doi: 10.1136/jitc-2021-002591 34330764 PMC8327843

[95] Simpson TR Li F Montalvo-Ortiz W Sepulveda MA Bergerhoff K Arce F. Fc-dependent depletion of tumor-infiltrating regulatory T cells co-defines the efficacy of anti-CTLA-4 therapy against melanoma. J Exp Med (2013) 210:1695–710. doi: 10.1084/jem.20130579 PMC3754863 23897981

[96] Arce Vargas F Furness AJS Litchfield K Joshi K Rosenthal R Ghorani E. Fc effector function contributes to the activity of human anti-CTLA-4 antibodies. Cancer Cell (2018) 33:649–663.e4. doi: 10.1016/j.ccell.2018.02.010 29576375 PMC5904288

[97] Hamid O Schmidt H Nissan A Ridolfi L Aamdal S Hansson J. A prospective phase II trial exploring the association between tumor microenvironment biomarkers and clinical activity of ipilimumab in advanced melanoma. J Transl Med (2011) 9:204. doi: 10.1186/1479-5876-9-204 22123319 PMC3239318

[98] Sharma A Subudhi SK Blando J Scutti J Vence L Wargo J. Anti-CTLA-4 immunotherapy does not deplete FOXP3+ Regulatory T cells (Tregs) in human cancers. Clin Cancer Res (2019) 25:1233–8. doi: 10.1158/1078-0432.CCR-18-0762 PMC6348141 30054281

[99] Rhodes KR Green JJ. Nanoscale artificial antigen presenting cells for cancer immunotherapy. Mol Immunol (2018) 98:13–8. doi: 10.1016/j.molimm.2018.02.016 PMC6084459 29525074

[100] Steenblock ER Fadel T Labowsky M Pober JS Fahmy TM. An artificial antigen-presenting cell with paracrine delivery of IL-2 impacts the magnitude and direction of the T cell response. J Biol Chem (2011) 286:34883–92. doi: 10.1074/jbc.M111.276329 PMC3186438 21849500

[101] Fadel TR Sharp FA Vudattu N Ragheb R Garyu J Kim D. A carbon nanotube-polymer composite for T-cell therapy. Nat Nanotechnol (2014) 9:639–47. doi: 10.1038/nnano.2014.154 25086604

[102] Perica K Tu A Richter A Bieler JG Edidin M Schneck JP. Magnetic field-induced T cell receptor clustering by nanoparticles enhances T cell activation and stimulates antitumor activity. ACS Nano (2014) 8:2252–60. doi: 10.1021/nn405520d PMC4004316 24564881

[103] Rhodes KR Isser A Hickey JW Ben-Akiva E Meyer RA Kosmides AK. Biodegradable cationic polymer blends for fabrication of enhanced artificial antigen presenting cells to treat melanoma. ACS Appl Mater Interfaces (2021) 13:7913–23. doi: 10.1021/acsami.0c19955 PMC8034558 33573372

[104] Sunshine JC Perica K Schneck JP. Green JJ. Particle shape dependence of CD8+ T cell activation by artificial antigen presenting cells. Biomaterials (2014) 35:269–77. doi: 10.1016/j.biomaterials.2013.09.050 PMC3902087 24099710

[105] Cheung AS Zhang DKY Koshy ST Mooney DJ. Scaffolds that mimic antigen-presenting cells enable ex vivo expansion of primary T cells. Nat Biotechnol (2018) 36:160–9. doi: 10.1038/nbt.4047 PMC5801009 29334370

[106] Kinoh H Quader S Shibasaki H Liu X Maity A Yamasoba T. Translational nanomedicine boosts anti-PD1 therapy to eradicate orthotopic PTEN-negative glioblastoma. ACS Nano (2020) 14:10127–40. doi: 10.1021/acsnano.0c03386 32806051

[107] Catania G Rodella G Vanvarenberg K Préat V Malfanti A. Combination of hyaluronic acid conjugates with immunogenic cell death inducer and CpG for glioblastoma local chemo-immunotherapy elicits an immune response and induces long-term survival. Biomaterials (2023) 294:122006. doi: 10.1016/j.biomaterials.2023.122006 36701998

[108] Teo PY Yang C Whilding LM Parente-Pereira AC Maher J George AJT. Ovarian cancer immunotherapy using PD-L1 siRNA targeted delivery from folic acid-functionalized polyethylenimine: strategies to enhance T cell killing. Adv Healthc Mater (2015) 4:1180–9. doi: 10.1002/adhm.201500089 25866054

[109] Yang T Mochida Y Liu X Zhou H Xie J Anraku Y. Conjugation of glucosylated polymer chains to checkpoint blockade antibodies augments their efficacy and specificity for glioblastoma. Nat BioMed Eng (2021) 5:1274–87. doi: 10.1038/s41551-021-00803-z 34635819

[110] Lin Y-X Wang Y Ding J Jiang A Wang J Yu M. Reactivation of the tumor suppressor PTEN by mRNA nanoparticles enhances antitumor immunity in preclinical models. Sci Transl Med (2021) 13:eaba9772. doi: 10.1126/scitranslmed.aba9772 34162754 PMC8284983

[111] Lei C Liu P Chen B Mao Y Engelmann H Shin Y. Local release of highly loaded antibodies from functionalized nanoporous support for cancer immunotherapy. J Am Chem Soc (2010) 132:6906–7. doi: 10.1021/ja102414t PMC2874126 20433206

[112] Roeven MWH Hobo W van der Voort R Fredrix H Norde WJ Teijgeler K. Efficient nontoxic delivery of PD-L1 and PD-L2 siRNA into dendritic cell vaccines using the cationic lipid SAINT-18. J Immunother (2015) 38:145–54. doi: 10.1097/CJI.0000000000000071 25839440

[113] Wang X Li X Ito A Watanabe Y Sogo Y Tsuji NM. Stimulation of *in vivo* antitumor immunity with hollow mesoporous silica nanospheres. Angew Chem Int Ed Engl (2016) 55:1899–903. doi: 10.1002/anie.201506179 26404897

[114] Ye Y Wang J Hu Q Hochu GM Xin H Wang C. Synergistic transcutaneous immunotherapy enhances antitumor immune responses through delivery of checkpoint inhibitors. ACS Nano (2016) 10:8956–63. doi: 10.1021/acsnano.6b04989 27599066

[115] Xiao Y Zhu T Zeng Q Tan Q Jiang G Huang X. Functionalized biomimetic nanoparticles combining programmed death-1/programmed death-ligand 1 blockade with photothermal ablation for enhanced colorectal cancer immunotherapy. Acta Biomater (2023) 157:451–66. doi: 10.1016/j.actbio.2022.11.043 36442821

[116] Feig C Jones JO Kraman M Wells RJB Deonarine A Chan DS. Targeting CXCL12 from FAP-expressing carcinoma-associated fibroblasts synergizes with anti-PD-L1 immunotherapy in pancreatic cancer. Proc Natl Acad Sci U.S.A. (2013) 110:20212–7. doi: 10.1073/pnas.1320318110 PMC3864274 24277834

[117] Tang T Huang X Zhang G Hong Z Bai X Liang T. Advantages of targeting the tumor immune microenvironment over blocking immune checkpoint in cancer immunotherapy. Signal Transduct Target Ther (2021) 6:72. doi: 10.1038/s41392-020-00449-4 33608497 PMC7896069

[118] Miao L Li J Liu Q Feng R Das M Lin CM. Transient and local expression of chemokine and immune checkpoint traps to treat pancreatic cancer. ACS Nano (2017) 11:8690–706. doi: 10.1021/acsnano.7b01786 PMC5961942 28809532

[119] Reichel D Tripathi M Perez JM. Biological effects of nanoparticles on macrophage polarization in the tumor microenvironment. Nanotheranostics (2019) 3:66–88. doi: 10.7150/ntno.30052 30662824 PMC6328304

[120] Qian Y Qiao S Dai Y Xu G Dai B Lu L. Molecular-targeted immunotherapeutic strategy for melanoma *via* dual-targeting nanoparticles delivering small interfering RNA to tumor-associated macrophages. ACS Nano (2017) 11:9536–49. doi: 10.1021/acsnano.7b05465 28858473

[121] Wang Y Lin Y-X Qiao S-L An H-W Ma Y Qiao Z-Y. Polymeric nanoparticles promote macrophage reversal from M2 to M1 phenotypes in the tumor microenvironment. Biomaterials (2017) 112:153–63. doi: 10.1016/j.biomaterials.2016.09.034 27768970

[122] Henry CJ Ornelles DA Mitchell LM Brzoza-Lewis KL Hiltbold EM. IL-12 produced by dendritic cells augments CD8+ T cell activation through the production of the chemokines CCL1 and CCL17. J Immunol (2008) 181:8576–84. doi: 10.4049/jimmunol.181.12.8576 PMC2716729 19050277

[123] Watkins SK Egilmez NK Suttles J Stout RD. IL-12 rapidly alters the functional profile of tumor-associated and tumor-infiltrating macrophages in *vitro* and in *vivo* . J Immunol (2007) 178:1357–62. doi: 10.4049/jimmunol.178.3.1357 17237382

[124] Chaudhuri AA So AY-L Sinha N Gibson WSJ Taganov KD O’Connell RM. MicroRNA-125b potentiates macrophage activation. J Immunol (2011) 187:5062–8. doi: 10.4049/jimmunol.1102001 PMC3208133 22003200

[125] Parayath NN Parikh A Amiji MM. Repolarization of tumor-associated macrophages in a genetically engineered nonsmall cell lung cancer model by intraperitoneal administration of hyaluronic acid-based nanoparticles encapsulating microRNA-125b. Nano Lett (2018) 18:3571–9. doi: 10.1021/acs.nanolett.8b00689 29722542

[126] Zanganeh S Hutter G Spitler R Lenkov O Mahmoudi M Shaw A. Iron oxide nanoparticles inhibit tumour growth by inducing pro-inflammatory macrophage polarization in tumour tissues. Nat Nanotechnol (2016) 11:986–94. doi: 10.1038/nnano.2016.168 PMC5198777 27668795

[127] Parayath NN Gandham SK Leslie F Amiji MM. Improved anti-tumor efficacy of paclitaxel in combination with MicroRNA-125b-based tumor-associated macrophage repolarization in epithelial ovarian cancer. Cancer Lett (2019) 461:1–9. doi: 10.1016/j.canlet.2019.07.002 31288064 PMC6682447

[128] Qian B-Z Pollard JW. Macrophage diversity enhances tumor progression and metastasis. Cell (2010) 141:39–51. doi: 10.1016/j.cell.2010.03.014 20371344 PMC4994190

[129] Serafini P Meckel K Kelso M Noonan K Califano J Koch W. Phosphodiesterase-5 inhibition augments endogenous antitumor immunity by reducing myeloid-derived suppressor cell function. J Exp Med (2006) 203:2691–702. doi: 10.1084/jem.20061104 PMC2118163 17101732

[130] Wiers KM Lathers DM Wright MA Young MR. Vitamin D3 treatment to diminish the levels of immune suppressive CD34+ cells increases the effectiveness of adoptive immunotherapy. J Immunother (2000) 23:115–24. doi: 10.1097/00002371-200001000-00014 10687144

[131] Hengesbach LM Hoag KA. Physiological concentrations of retinoic acid favor myeloid dendritic cell development over granulocyte development in cultures of bone marrow cells from mice. J Nutr (2004) 134:2653–9. doi: 10.1093/jn/134.10.2653 15465762

[132] Ko JS Zea AH Rini BI Ireland JL Elson P Cohen P. Sunitinib mediates reversal of myeloid-derived suppressor cell accumulation in renal cell carcinoma patients. Clin Cancer Res (2009) 15:2148–57. doi: 10.1158/1078-0432.CCR-08-1332 19276286

[133] Lu P Yu B Xu J. Cucurbitacin B regulates immature myeloid cell differentiation and enhances antitumor immunity in patients with lung cancer. Cancer Biother Radiopharm (2012) 27(8):495–503. doi: 10.1089/cbr.2012.1219 22746287

[134] Suzuki E Kapoor V Jassar AS Kaiser LR Albelda SM. Gemcitabine selectively eliminates splenic Gr-1+/CD11b+ myeloid suppressor cells in tumor-bearing animals and enhances antitumor immune activity. Clin Cancer Res (2005) 11:6713–21. doi: 10.1158/1078-0432.CCR-05-0883 16166452

[135] Le HK Graham L Cha E Morales JK Manjili MH Bear HD. Gemcitabine directly inhibits myeloid derived suppressor cells in BALB/c mice bearing 4T1 mammary carcinoma and augments expansion of T cells from tumor-bearing mice. Int Immunopharmacol (2009) 9:900–9. doi: 10.1016/j.intimp.2009.03.015 19336265

[136] Plebanek MP Bhaumik D Bryce PJ Thaxton CS. Scavenger receptor type B1 and lipoprotein nanoparticle inhibit myeloid-derived suppressor cells. Mol Cancer Ther (2018) 17:686–97. doi: 10.1158/1535-7163.MCT-17-0981 PMC5935575 29282300

[137] Kong M Tang J Qiao Q Wu T Qi Y Tan S. Biodegradable hollow mesoporous silica nanoparticles for regulating tumor microenvironment and enhancing antitumor efficiency. Theranostics (2017) 7:3276–92. doi: 10.7150/thno.19987 PMC5595131 28900509

[138] Curti B Daniels GA McDermott DF Clark JI Kaufman HL Logan TF. Improved survival and tumor control with Interleukin-2 is associated with the development of immune-related adverse events: data from the PROCLAIMSM registry. J Immunother Cancer (2017) 5:102. doi: 10.1186/s40425-017-0307-5 29254506 PMC5735508

[139] Hamson EJ Keane FM Tholen S Schilling O Gorrell MD. Understanding fibroblast activation protein (FAP): substrates, activities, expression and targeting for cancer therapy. Proteomics Clin Appl (2014) 8:454–63. doi: 10.1002/prca.201300095 24470260

[140] Neesse A Michl P Frese KK Feig C Cook N Jacobetz MA. Stromal biology and therapy in pancreatic cancer. Gut (2011) 60:861–8. doi: 10.1136/gut.2010.226092 20966025

[141] Ernsting MJ Hoang B Lohse I Undzys E Cao P Do T. Targeting of metastasis-promoting tumor-associated fibroblasts and modulation of pancreatic tumor-associated stroma with a carboxymethylcellulose-docetaxel nanoparticle. J Control Release (2015) 206:122–30. doi: 10.1016/j.jconrel.2015.03.023 PMC4409566 25804872

[142] Liu C Lai H Chen T. Boosting natural killer cell-based cancer immunotherapy with selenocystine/transforming growth factor-beta inhibitor-encapsulated nanoemulsion. ACS Nano (2020) 14:11067–82. doi: 10.1021/acsnano.9b10103 32806028

[143] Lai H Zeng D Liu C Zhang Q Wang X Chen T. Selenium-containing ruthenium complex synergizes with natural killer cells to enhance immunotherapy against prostate cancer *via* activating TRAIL/FasL signaling. Biomaterials (2019) 219:119377. doi: 10.1016/j.biomaterials.2019.119377 31374478

[144] Hou L Liu Q Shen L Liu Y Zhang X Chen F. Nano-delivery of fraxinellone remodels tumor microenvironment and facilitates therapeutic vaccination in desmoplastic melanoma. Theranostics (2018) 8:3781–96. doi: 10.7150/thno.24821 PMC6071534 30083259

[145] Hu T Gong H Xu J Huang Y Wu F He Z. Nanomedicines for overcoming cancer drug resistance. Pharmaceutics (2022) 14:1606. doi: 10.3390/pharmaceutics14081606 36015232 PMC9412887

[146] Dietl K Renner K Dettmer K Timischl B Eberhart K Dorn C. Lactic acid and acidification inhibit TNF secretion and glycolysis of human monocytes. J Immunol (2010) 184:1200–9. doi: 10.4049/jimmunol.0902584 20026743

[147] Singer K Kastenberger M Gottfried E Hammerschmied CG Büttner M Aigner M. Warburg phenotype in renal cell carcinoma: high expression of glucose-transporter 1 (GLUT-1) correlates with low CD8(+) T-cell infiltration in the tumor. Int J Cancer (2011) 128:2085–95. doi: 10.1002/ijc.25543 20607826

[148] Bonatelli M Silva ECA Cárcano FM Zaia MG Lopes LF Scapulatempo-Neto C. The warburg effect is associated with tumor aggressiveness in testicular germ cell tumors. Front Endocrinol (Lausanne) (2019) 10:417. doi: 10.3389/fendo.2019.00417 31316469 PMC6610306

[149] Chang C-H Qiu J O’Sullivan D Buck MD Noguchi T Curtis JD. Metabolic competition in the tumor microenvironment is a driver of cancer progression. Cell (2015) 162:1229–41. doi: 10.1016/j.cell.2015.08.016 PMC4864363 26321679

[150] Platten M Wick W Van den Eynde BJ. Tryptophan catabolism in cancer: beyond IDO and tryptophan depletion. Cancer Res (2012) 72:5435–40. doi: 10.1158/0008-5472.CAN-12-0569 23090118

[151] Ikonen E. Cellular cholesterol trafficking and compartmentalization. Nat Rev Mol Cell Biol (2008) 9:125–38. doi: 10.1038/nrm2336 18216769

[152] Jakobsson T Treuter E Gustafsson J-Å Steffensen KR. Liver X receptor biology and pharmacology: new pathways, challenges and opportunities. Trends Pharmacol Sci (2012) 33:394–404. doi: 10.1016/j.tips.2012.03.013 22541735

[153] Li J Gu D Lee SS-Y Song B Bandyopadhyay S Chen S. Abrogating cholesterol esterification suppresses growth and metastasis of pancreatic cancer. Oncogene (2016) 35:6378–88. doi: 10.1038/onc.2016.168 PMC5093084 27132508

[154] Yang W Bai Y Xiong Y Zhang J Chen S Zheng X. Potentiating the antitumour response of CD8(+) T cells by modulating cholesterol metabolism. Nature (2016) 531:651–5. doi: 10.1038/nature17412 PMC4851431 26982734

[155] Thakur N Thakur S Chatterjee S Das J Sil PC. Nanoparticles as smart carriers for enhanced cancer immunotherapy. Front Chem (2020) 8:597806. doi: 10.3389/fchem.2020.597806 33409265 PMC7779678

